# The customer satisfaction model in the mobile telecommunications sector after Covid-19 pandemic

**DOI:** 10.1371/journal.pone.0317093

**Published:** 2025-01-27

**Authors:** Petr Suchanek, Natalia Bucicova

**Affiliations:** 1 Department of Business Economics, Mendel University in Brno Faculty of Business and Economics, Brno, Czech Republic; 2 Department of Business Economics and Management, Masaryk University Faculty of Economics and Administration, Brno, Czech Republic; Aalborg University, DENMARK

## Abstract

The subject of this paper is modeling customer satisfaction in the mobile telecommunication industry following the Covid-19 pandemic. Based on standard customer satisfaction models, a specialized model tailored for the mobile telecommunication industry has been developed to account for its unique characteristics, including market concentration. This model was created within the Slovakian context using the Structural Equation Modelling method. The respondents were customers of all mobile operators in this market. The model revealed a positive relationship between image and perceived service quality and a negative relationship between customer expectations and perceived service value. However, it was not possible to demonstrate a relationship between image and customer loyalty or between customer expectations and customer satisfaction. Therefore, it seems that the factors influencing customer satisfaction in the telecommunications sector of an emerging EU economy differ from those in other sectors and economies in the post-Covid-19 context.

## 1 Introduction

The mobile telecommunication services industry has undergone tremendous development in recent years [1–3], with continuous and exponential advancements in mobile technology [4]. Consequently, it is very challenging for companies operating in this concentrated industry to maintain a competitive advantage [5]. Therefore, customer satisfaction and loyalty are crucial for sustaining a company’s competitive edge in this industry [2].

Customer satisfaction modeling has been extensively addressed by various authors in the field of mobile telecommunications [6–14], including within the Slovak context [15]. However, the entire industry, and the use of mobile phones, has changed significantly due to the Covid-19 pandemic [16–18], which has led to structural changes [19]. These changes could also impact customer satisfaction, as seen in other sectors such as hospitality [20], mobile banking [21], hotel services [22], and mobile wallet services [23].

Research on customer satisfaction in the mobile telecommunications industry in the aftermath of the Covid-19 pandemic is still lacking. However, it can be assumed that these changes will also be reflected in the modeling of customer satisfaction in this industry. The relationships between the variables (factors) in the model may differ, as observed in other industries [20–23]. This, in turn, would have implications for managers’ decision-making; without the right information, they cannot make the right decisions. Consequently, firms’ competitiveness could decline if managerial decisions are incorrect and customer satisfaction and loyalty decrease [24].

The aim of this paper is to clarify the relationships between the modeled variables in the customer satisfaction model. This includes determining whether these relationships are positive or negative and identifying any changes compared to the period before the Covid-19 pandemic.

The structure of the article is as follows. After the Introduction, there is a section devoted to the Theoretical framework, namely the definition of customer satisfaction, including the ways of its measurement and the models that can be used for this measurement, which is followed by the definition of the factors that are commonly used in customer satisfaction modelling and the definition of the telecommunications industry. The next section is devoted to Hypothesis development and conceptual model, where hypotheses are defined using the literature to represent the relationships between the factors under investigation within a comprehensive customer satisfaction model. This is followed by the Materials and methods section, which is devoted to the construction of the questionnaire used, the statistical tools employed and the characteristics of the research sample. This is followed by a section on Results and then sections on Discussion and Conclusion.

## 2 Theoretical background

### 2.1 Customer satisfaction

Customer satisfaction can be defined in various ways. One of the most cited definitions conceptualizes customer satisfaction as a post-consumption evaluation of a product or service [25]. Perhaps the most comprehensive definition, consistent with the aforementioned concept, is provided by Giese & Cote [26]: “Customer satisfaction is a cumulative affective response of varying intensity with a time-specific destination and limited duration focused on core aspects of product acquisition and/or consumption.”

The literature suggests two types of satisfaction: transactional and cumulative (or total) satisfaction [27]. Transactional satisfaction is based on the evaluation of a single purchase, is narrowly focused on the product purchased, and is short-term [27]. This type of satisfaction will not be analyzed further as it pertains only to a single purchase—applicable to customers who bought the product more or less accidentally and did not buy it again. This scenario does not apply to telecommunication services, which customers use on a long-term basis and where they change operators over an extended period.

Cumulative customer satisfaction, on the other hand, can be defined as the overall purchase experience, or general satisfaction [28, 29]. Cumulative satisfaction is understood as long-term satisfaction based on the customer’s overall experience with the product and repeat purchases. This type of satisfaction may also include an evaluation of the seller, encompassing both the manufacturer and the retailer. Cumulative customer satisfaction is crucial for forming and maintaining the long-term relationship between the company and the customer. It is determined by the performance of various specific components or attributes [30].

Customer satisfaction is not static because consumption goals change [31] and needs and competitive forces are dynamic [32]. As Kanji & Wallace [33] point out, businesses must achieve continuous improvement in all aspects of their operations to achieve customer satisfaction. Given that customer satisfaction is constantly evolving, the question arises as to when or how often to measure it. Research on the frequency of feedback, including customer satisfaction surveys, and its effectiveness is inconclusive [34]. Thus, it cannot be definitively stated that increasing the frequency of feedback improves the product provided. Although authors disagree on the optimal frequency of feedback, they agree on its regularity—feedback, including customer satisfaction, should be collected regularly [34, 35].

Customer satisfaction can be conceptualized either as a single, though multidimensional, factor or as a structural model comprising several sub-factors that collectively influence overall satisfaction. The single-factor approach is typically employed when studying complex relationships among various variables, where customer satisfaction is just one aspect among many [36–39]. Conversely, the structural model of customer satisfaction is utilized when the focus is directly on understanding satisfaction and the specific factors that impact it [40–42].

Within the framework of customer satisfaction as a single factor, customer expectations are consistently recognized as a crucial component [37]. Additional variables such as competitiveness, loyalty, perceived value, perceived quality, image, and product familiarity complement these expectations within the customer satisfaction construct. These variables, including customer expectations, are typically part of a complex, multifactorial model of customer satisfaction where each represents distinct constructs [36, 37]. In this research, the authors adopted the multifactorial approach, constructing a comprehensive model to explore customer satisfaction.

Within the complex and multifactorial model, customer satisfaction represents a distinct factor known as general customer satisfaction, crucial for the sustainable development of enterprises [43]. This factor serves as an indicator that evaluates both past and present performance, providing a foundation for future development [44]. According to research by Türkyilmaz & Özkan [7], Balaji [45], and Jallow [8], general customer satisfaction comprises three key components: overall satisfaction, meeting customer expectations, and company performance relative to competitors [8]. Türkyilmaz et al. [9], focusing on the telecommunications industry, also identified these three variables, but they modified the comparison aspect from competitors to an ideal standard. Strenitzer & Gaña [15], on the other hand, included two variables: meeting service expectations and overall satisfaction with the mobile service provider.

Several comprehensive models have been developed in the telecommunications industry to measure customer satisfaction. These include models based on ACSI [6, 7, 10, 12] and ECSI [8, 9, 11, 15], which quantify overall satisfaction, as well as complex models focusing on interrelationships between factors [13, 14].

In our research, we concentrate on quantifying these interrelationships using the ECSI model, which is currently one of the most comprehensive in terms of the number of factors and interrelationships. This model is particularly service-oriented. The following section outlines the different factors used in our model, excluding customer satisfaction, which has been characterized earlier.

### 2.2 Factors of customer satisfaction

The **image** of a business encompasses the associations that customers form with its brand or name [46]. It includes the impressions, feelings, and knowledge customers acquire through interactions with the business [43]. Image is considered a fundamental variable of customer satisfaction [47]. Research indicates that image influences customer loyalty, perceived value, overall satisfaction [48, 49], and ultimately, the company’s market position [43].

A company’s image reflects perceptions of its trustworthiness, professionalism, innovation, and societal contribution, enhancing its standing not only in the market but also in the eyes of customers [9]. From a marketing perspective, image encompasses variables related to the brand, logo, and physical premises, shaping the overall perception of the company [43]. Saeidi et al. [50] suggest that a company’s image is also shaped by its capabilities and commitment to social responsibility.

In the telecommunications industry, various factors contribute to the creation of image. Türkyilmaz et al. [9] identify reliability, professionalism, contribution to society, customer relations, innovation, and progressiveness as key variables. Additionally, Jallow [8] includes user value, while Ansah [51] emphasizes credibility, social responsibility, and reputation for quality as important components. Strenitzer & Gaňa [15], in their study on the Slovak telecommunications market, specifically examined how factors such as the appearance of provider outlets, the design of employee uniforms, logo recognition among customers, and the clarity of promotional materials, brochures, and websites contribute to shaping the image of telecom service providers.

**Customer expectations** in business contexts provide insights into what customers anticipate from a company regarding product quality and quantity. These expectations can stem from prior experiences with a company’s products or services [52] or from perceived capabilities to fulfill various objectives during a purchase decision [53]. In the telecommunications sector, Türkyilmaz et al. [9] studied customer expectations in terms of personal need satisfaction, overall quality, product quality, and service quality. Strenitzer & Gaňa [15] expanded on these factors by including expectations related to speed and staff readiness.

**Perceived** (service) **quality**, as defined by Parasuraman et al. [54], refers to a customer’s overall judgment or attitude regarding the superiority of a service compared to alternatives. This perception is not merely based on the service’s attributes but rather on the customer’s feelings and memories, reflecting the satisfaction derived from the service experience [55]. Thus, perceived service quality represents how reliably and readily available customers perceive the service to be [56].

In the telecommunications industry, the variables examined for perceived quality vary significantly among studies. Türkyilmaz et al. [9] based their model on factors defined by Fornell et al. [44], which include overall quality, product quality, and service quality. They further added customer service quality and the appropriateness of quality to customer intentions. Strenitzer & Gaňa [15] expanded their focus to nine factors: overall perceived quality of products and services, quality of providers’ access to new technologies, quality of customer service and support, quality of availability of branches and customer service lines, quality of the selection of products and services offered, reliability and accuracy of services provided, clarity and transparency of provided information, competence of staff, and ability to customize service.

In contrast, Reis et al. [11] identified six distinct factors: leadership, strategy, people, processes, results and performance, and partnership, which they associated with perceived quality in the telecommunications sector. These varied approaches illustrate the complexity and breadth of factors considered when assessing perceived quality in telecommunications, reflecting different aspects of service delivery, customer interactions, and organizational capabilities.

**Perceived value** in the context of customer assessment refers to the psychological evaluation of a product or service based on the expectations associated with it [53]. Essentially, it reflects how customers perceive the benefits received relative to their expectations, determining whether the service provided meets their anticipated utility [43]. Perceived value is recognized as a significant driver of competitiveness [57] and is often considered in relation to pricing strategies [53, 58]. Assessing the price-quality ratio allows for comparisons between companies [59].

In their research, Türkyilmaz et al. [9] focused on two specific aspects: the price-performance ratio and the performance-price ratio. Strenitzer & Gaňa [15] explored multiple factors related to perceived value, including evaluations of calling services in terms of pricing and parameters, assessments of service quality relative to pricing, evaluations of satisfaction with the quantity and quality of benefits and discounts offered, and the appropriateness of the company’s marketing communications. These various factors underscore the multifaceted nature of perceived value in telecommunications, highlighting the importance of both service quality and pricing strategies in shaping customer perceptions and satisfaction.

**Loyalty** in the context of customer behavior is defined as the tendency to repeatedly purchase services from the same company [60]. This repeated patronage reflects a commitment on the part of customers to continue choosing the same service provider [61]. Ensuring customer loyalty is crucial for sustaining future revenue and reducing the risk of customer turnover, particularly in the face of declining service quality [52]. However, maintaining loyalty is challenging in today’s competitive business environment [43].

Various metrics are used to measure loyalty. Common approaches include assessing customers’ intentions to make repeat purchases, their willingness to tolerate changes in pricing or service offerings, and their likelihood to recommend the service provider to others [52]. In the Turkish telecommunications market, loyalty has been examined through inquiries into repeat purchase intentions, recommendations to acquaintances, and tolerance for price fluctuations [9]. Similarly, in the Slovak mobile operator market, loyalty research has focused on customers’ willingness to remain with their provider despite competitors offering lower prices for similar services, their inclination to recommend the provider to friends or family, and their future commitment to stay with the same provider [15]. These loyalty metrics provide insights into customer commitment and satisfaction, crucial for telecommunications companies aiming to retain their customer base amidst competitive pressures.

### 2.3 The mobile telecommunications sector

Telecommunications, particularly mobile telecommunications, play a crucial role in facilitating information transfer and connectivity in modern society [62]. They enable simultaneous communication in various forms—spoken or written—while adding value through mediation, monitoring, and maintaining transmission [63]. As part of the broader electronic communications sector, mobile telecommunications utilize electronic communication networks to function effectively [62]. This industry is vital to the economy, influencing various activities and often subject to government regulation [64, 65].

Mobile telecommunication services have become integral to daily life, particularly highlighted during the Covid-19 pandemic, where reliance on information technology surged [3]. These services generally fall into two categories: residential services (such as fixed Internet and TV) and mobile services encompassing voice (calls) and data (mobile Internet) services [66]. For our paper’s scope, we focus specifically on mobile services provided by mobile operators, excluding other segments of the telecommunications sector. This focused approach allows for a detailed examination of customer satisfaction and related factors within the mobile telecommunications industry, addressing specific nuances and dynamics unique to this sector.

The mobile telecommunications market has seen remarkable growth and transformation over the past decade, reflecting its pivotal role in global connectivity. Research from a decade ago indicated that three-quarters of the global population had access to a mobile phone [67], a figure that has since risen to over 84% [2]. This expansion underscores the market’s immense potential, with a key challenge being customer retention and loyalty [2, 68].

Historically, the telecommunications sector has often been dominated by monopolies due to significant market barriers such as high switching costs and infrastructure investments [69, 70]. These barriers were largely rooted in analogue technologies that operated on a national scale. However, since then, the industry has undergone substantial evolution and infrastructure reconstruction [67, 71].

Technological advancements have played a pivotal role in reshaping the telecommunications landscape. New generations of mobile networks, such as 4G and 5G, have brought about greater network capacity, faster data transmission speeds, higher quality voice services, and other innovations [4, 72, 73]. This ongoing technological progress continues to redefine service capabilities and user experiences in the mobile telecommunications sector.

In summary, the mobile telecommunications industry has evolved significantly, driven by technological advancements and expanding global accessibility, presenting both opportunities and challenges for stakeholders aiming to enhance customer satisfaction and retention in an increasingly competitive market.

The mobile telecommunications market in the European Union, including Slovakia, is characterized as an oligopoly, where a small number of operators dominate [74–77]. This oligopolistic structure arises due to several factors that create barriers to entry and limit competition. One primary reason for the low competition is the substantial technological and financial barriers to entering the market [63]. Establishing and maintaining telecommunications infrastructure, along with deploying services, require significant investments. Additionally, spectrum allocation and regulatory constraints further complicate entry into the market, contributing to what can be termed as a natural monopoly in certain aspects [78].

As a result, new entrants face challenges in competing with established operators who have already invested heavily in infrastructure and hold significant market share [79]. This concentration of market power leads to a competitive landscape typically dominated by three to four major companies in most EU markets, including Slovakia [5, 69, 75]. In this oligopolistic environment, the competitive dynamics among operators are crucial for understanding market behaviors, pricing strategies, service innovations, and ultimately, their impact on customer satisfaction and loyalty in the mobile telecommunications sector.

In Slovakia, the telecommunications sector, specifically mobile electronic communications services, is dominated by four main providers [80]. These operators offer highly similar services and compete primarily on innovation, product and service quality, and pricing strategies. Despite their efforts to differentiate themselves in these areas, customer perception of the sector remains somewhat negative. Research has indicated that the telecommunications sector in Slovakia is perceived unfavorably by customers, ranking seventh out of ten sectors analyzed [81]. One of the key reasons for this perception is the perceived annual deterioration in products and services offered by mobile operators. This suggests that while operators strive to innovate and improve their offerings, customer satisfaction may be impacted by issues such as service quality fluctuations or perceived declines in service over time.

The competitive dynamics in an oligopolistic market like Slovakia’s telecommunications sector highlight the challenges and pressures faced by operators in balancing innovation, quality maintenance, pricing strategies, and customer satisfaction. Addressing these challenges effectively is crucial for enhancing customer perceptions and maintaining competitiveness in the market.

In assessing the quality of mobile electronic communication services, researchers distinguish between several levels: technological, functional, social, and environmental. These levels provide a comprehensive framework for evaluating different aspects of service quality.

1. **Technological Level**: This level is assessed based on objective criteria such as the process of service introduction, operational reliability, connectivity with other services, technical support, and overall technical performance [82].
2. **Functional Level**: Unlike the technological level, the functional level of service quality is evaluated based on subjective customer criteria. This includes factors such as the image of the service provider, customer support effectiveness and competence of staff, marketing efforts of the company, and other aspects that directly impact the customer experience [82].
3. **Social Level**: This level incorporates aspects related to corporate social responsibility (CSR) and communication in this area. It assesses how mobile operators engage with social issues, support community initiatives, and communicate their CSR efforts to stakeholders [83].
4. **Environmental Level**: The environmental level focuses on environmental responsibility, including e-waste management, efforts to protect nature and biodiversity, and communication about these environmental initiatives [83].

Given the broad scope of these areas, your research focuses specifically on the functional level of service quality. This approach allows for a deeper exploration of customer-centric factors that influence perceptions of service quality and satisfaction, such as customer service effectiveness and the provider’s overall image. By concentrating on the functional level, your study aims to provide insights into how these subjective criteria impact customer satisfaction and loyalty within the mobile telecommunications sector.

## 3 Hypothesis development and conceptual model

**Fig 1:**
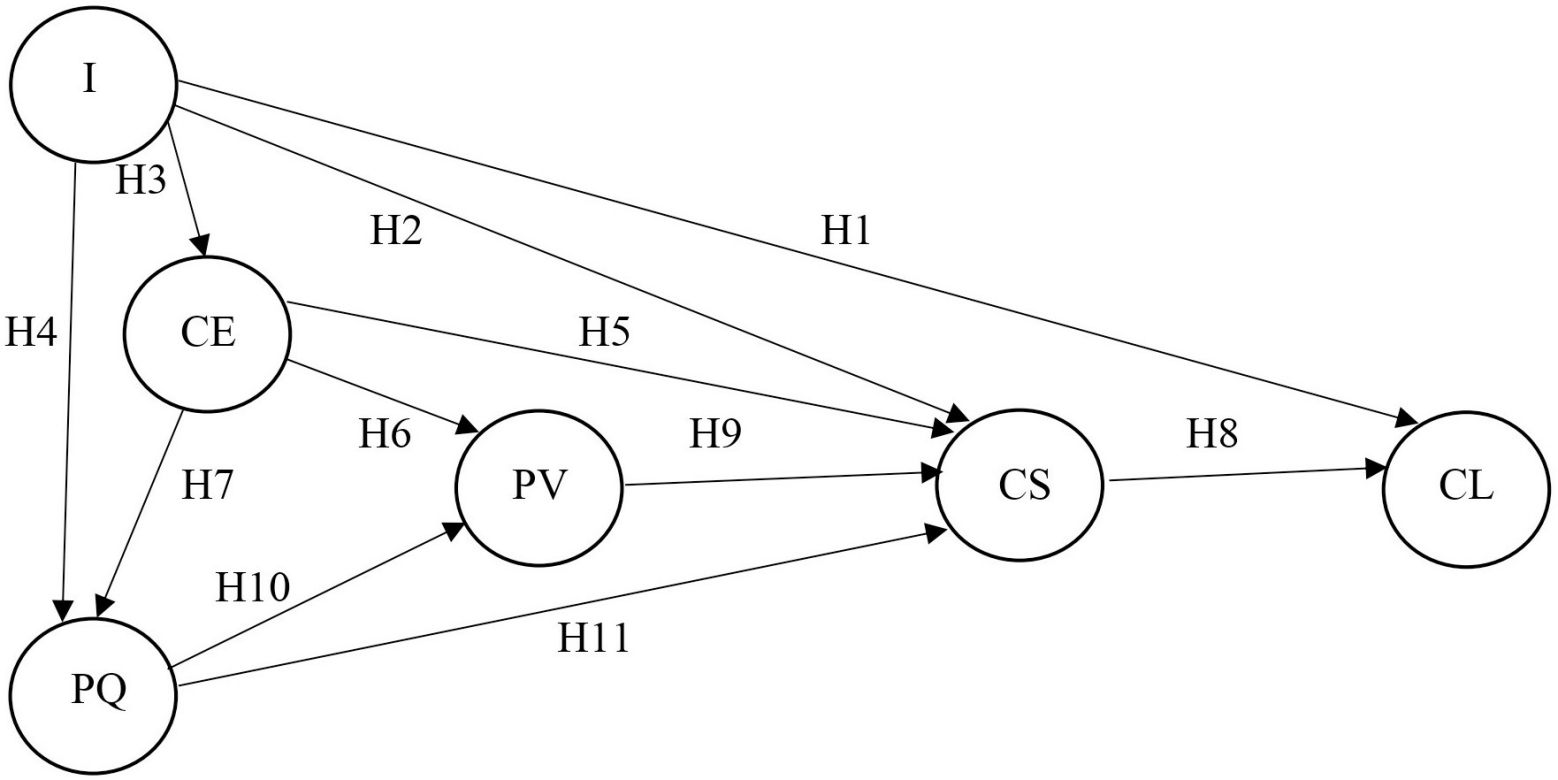
Customer satisfaction model in the mobile telecommunications industry. Source: own research.

**Fig 2:**
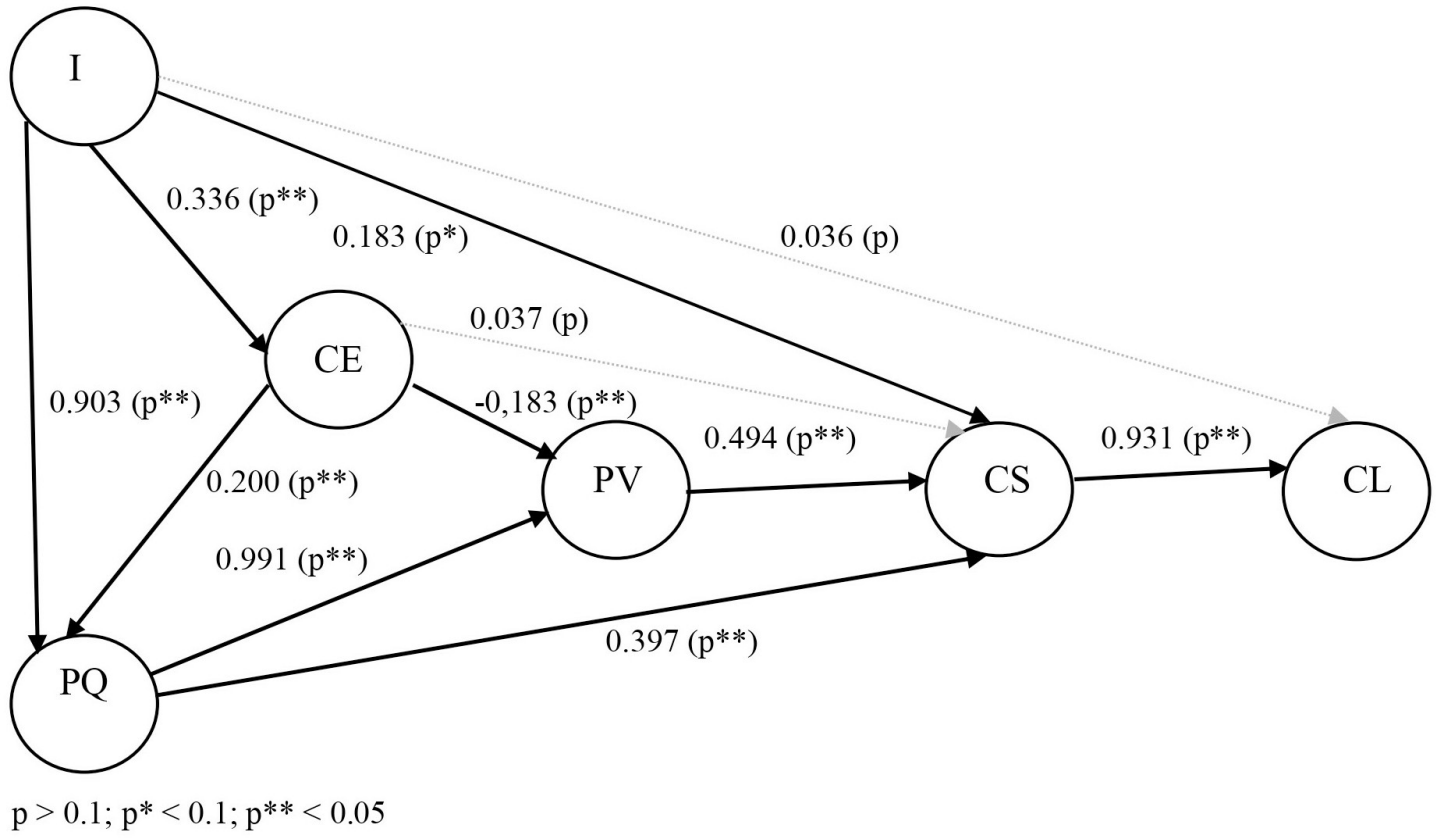
The resulting model of customer satisfaction in the telecommunications industry. Source: own research.

All examined relationships between the variables are defined by the hypotheses listed below. For clarity, these relationships and hypotheses are then visualised in the conceptual model presented in Figs 1 and 2.

Image is one of the important variables in customer satisfaction models focused on services [46, 84] and specifically on mobile telecommunications [15, 85, 86]. According to both Diputra & Yasa [86] and Yilmaz & Ari [84], image only affects customer satisfaction and not loyalty, while product quality affects image, which is also supported by the research of Babic-Hodovic et al. [87]. The influence of image on customer satisfaction is also confirmed by Strenitzer & Gaňa [15], while also confirming the influence of image on perceived quality (of product and service), as well as Gluhović [88]. The influence of image on customer satisfaction is also confirmed by Dam & Dam [46], and in addition confirms the influence on customer loyalty. The influence of image on customer satisfaction, customer loyalty and moreover on customer expectation is also confirmed by the research of Mohd Jamil et al. [85]. Thus, it is clear that image can be expected to be related with customer satisfaction, customer loyalty, product quality and customer expectations. Thus, based on the results of these researches, the following four hypotheses were formulated:

**H1:** Image is associated with customer satisfaction of mobile operators.
**H2:** Image is associated with mobile operators’ customers’ expectations.
**H3:** Image is associated with customer loyalty of mobile operators.
**H4:** Image is associated with perceived quality of mobile operators.

In the literature, there is no uniform view on the relationship between customer expectations and other variables. Some authors have found that customer expectations do not have a significant effect on customer satisfaction [48, 89, 90]. On the other hand, there are authors who argue that expectations have a direct and positive relationship with satisfaction [9, 10, 53, 91]. The literature also reports an ambiguous relationship between customer expectations and perceived value [44], which was found to be non-significant in the research of Jallow [8]. In contrast, the influence of customer expectations on perceived value (and also perceived quality and customer satisfaction) was demonstrated by Morgeson et al. [10]. Strenitzer & Gaňa [15] in their research demonstrated the influence of customer expectations on perceived quality (of both product and service). However, it is clear that a relationship between customer expectations on the one hand and customer satisfaction, perceived value and perceived quality on the other hand can be expected. Thus, based on the results of these researches, the following three hypotheses were formulated:

**H5:** Customer expectations are associated with customer satisfaction of mobile operators.
**H6:** Customer expectations are associated with the perceived quality of mobile operators.
**H7:** Customer expectations are associated with perceived value of mobile operators.

The influence of customer satisfaction on customer loyalty has been demonstrated in a number of studies [92–95] and also in the case of telecommunications [7, 15, 96, 97]. Thus, based on the results of these studies, the following hypothesis was formulated:

**H8:** Customer satisfaction is related to mobile operators’ customer loyalty.

Türkyilmaz & Ozkan [7] state that perceived value has a positive effect on customer satisfaction (in the case of telecommunications), which has been subsequently confirmed by Ali et al. [53], Mahmoud et al. [58] and Strenitzer & Gaňa [15]. Therefore, the following hypothesis focuses on this relationship:

**H9:** Perceived value is related to mobile operators’ customer satisfaction.

Research has shown that perceived service quality influences customer satisfaction [98, 99] and this is also the case in telecommunications [97, 100]. A number of studies have also shown that perceived quality has a positive influence on perceived value [44]. In the case of telecommunication services, the influence of perceived quality on perceived value and customer satisfaction has been demonstrated by Türkyilmaz & Ozkan [7] and Strenitzer & Gaňa [15]. It is therefore clear that a relationship between perceived quality and perceived value and perceived quality and customer satisfaction can be expected. Thus, based on the results of these researches, the following two hypotheses were formulated:

**H10:** Perceived quality is related to customer satisfaction of mobile operators.
**H11:** Perceived quality is related to perceived customer value of mobile operators.

## 4 Materials and methods

### 4.1 The questionnaire

The questionnaire was developed based on an analysis of several studies related to customer satisfaction [9, 15, 43, 52, 101, 102]. The questions are rated using a seven-point Likert scale, consistent with studies such as Rahman [103] in the telecommunications industry in Bangladesh, Strenitzer & Gaňa [15] in research on mobile operator customer satisfaction and loyalty in Slovakia, and Dachyar & Noviannei [104] in the telecommunications industry in Indonesia. The scale ranges from 1 (strongly disagree) to 7 (strongly agree). The questionnaire comprises twenty-seven closed-ended questions, including twenty questions measuring satisfaction with various variables and seven questions gathering demographic information. For detailed questions, refer to Tables 1 and 2.

**Table 1:** Questions on individual variables of the customer satisfaction model.

Variable	Question from the questionnaire	Scale
IM1	Mobile operator innovates services	1—strongly disagree 2—disagree 3- rather disagree 4- neither agree nor disagree 5- rather agree 6—agree 7- strongly agree
IM2	Employees have a professional approach to me	
IM3	Mobile operator engages in social responsibility activities	
EXP1	I expect quality of service provided by the mobile operator	
EXP2	I expect my needs to be met based on the expectations I have of the mobile operator	
EXP3	I expect the mobile operator’s staff to be prompt and ready to solve problems with the service provided	
PQ1	The mobile operator provides quality voice and data services	
PQ2	The mobile operator provides a quality mobile network and coverage	
PQ3	The mobile operator has a quality mobile application	
PQ4	The mobile operator provides quality customer support	
PQ5	The mobile operator has a dense network of branches	
PV1	The mobile operator’s services are affordable for me	
PV2	The mobile operator offers good value for money	
PV3	The price of mobile services with my operator is better than competitors	
CS1	Overall, I am satisfied with my mobile operator	
CS2	The mobile operator meets all my expectations	
CS3	Compared to possible ideal mobile services, I am satisfied with the ones I use	
CL1	I plan to purchase my mobile operator’s services again in the future	
CL2	I would recommend my mobile operator to family, friends and acquaintances	
CL3	I will continue to use my mobile operator’s services even if they increase prices	

Source: own research according to O´Loughlin & Coenders [52]; Türkyilmaz et al. [9]; Sabir et al. [101]; Rajendran & Suresh [102]; Strenitzerová & Gaňa [15]; Nguyen [43]

**Table 2:** Demographic questions.

Variable	Demographic characteristics	Answer options
I.	Which mobile operator do you use?	1 Orange Slovensko, a.s. 2 O2 Slovakia s.r.o. 3 Slovak Telekom, a.s. 4 SWAN Mobile, a.s. (4ka)
II.	What is your gender?	1 Man 2 Woman
III.	What is your age?	1 Pre-productive (0–14) 2 Productive (15–64) 3 Post-productive (65 and over)
IV.	Are you currently:	1 Employee 2 Unemployed 3 Student 4 Pensioner 5 On maternity/parental leave 6 Self-employed 7 Other
V.	What is your highest level of education?	1 None/ not yet completed 2 Basic 3 Secondary without diploma 4 Secondary school with diploma 5 Higher vocational 6 Higher education
VI.	What is your marital status?	1 Single 2 Married 3 Divorced 4 Widowed
VII.	Do you live in a village/town that has:	1 to 4 999 2 5 000–9 999 3 10 000–19 999 4 20 000–49 999 5 50 000–99 999 6 100 000 and more

Source: own research according to scitanie.sk. [105]

The next table shows the construction of questions focusing on demographic characteristics. The demographic questions have been taken and designed taking into account data from the Scitanie.sk website, which offers information about the 2021 census [105].

### 4.2 Statistical tools

Multicollinearity in the data was assessed using both the correlation matrix and the Variance Inflation Factor (VIF). According to Franke [106], correlations should ideally not exceed 0.8 to 0.9 to avoid significant multicollinearity; lower correlation values indicate lesser multicollinearity. Cheng et al. [107] suggest that VIF values above 10 indicate collinearity, with lower values indicating less severe collinearity.

Internal consistency was evaluated using Cronbach’s alpha. Generally, Cronbach’s alpha should fall between 0.7 and 0.9 [108], with the minimum acceptable value set at 0.65 [109]. Tavakol & Dennick [110] advise that values up to 0.95 are acceptable, though values approaching 1.0 may indicate redundancy among items in the scale.

To analyze the data collected from the questionnaire, Structural Equation Modeling (SEM) was employed, a method commonly utilized in customer satisfaction studies within the mobile telecommunications sector [111–114]. SEM incorporates path analysis to examine direct and indirect causal relationships among latent variables [115]. The strength of these relationships is quantified using regression coefficients [116].

A measurement model is integral to SEM and describes the associations between latent variables and their observable indicators [7]. Confirmatory Factor Analysis (CFA) is employed within the measurement model to assess individual constructs, determining how measurable variables contribute to latent variables with their respective factor loadings (regression coefficients) [116].

The validity of the measurement model was assessed for both convergence and discrimination. Discriminant validity was evaluated using the Average Variance Extracted (AVE) index, which ideally should exceed 0.5 [117]; however, a value above 0.4 is considered acceptable [118]. Convergent validity was assessed through the factor loadings of the constructs, with standardized regression coefficients expected to be at least 0.5 [119]. Reliability was evaluated using the Construct Reliability (CR) index, which should be minimally 0.7 [119].

Several fit indices were employed to assess the adequacy of the SEM model [115]: the Comparative Fit Index (CFI), which should be at least 0.9; the Tucker-Lewis Index (TLI), also expected to be at least 0.9; the Standardized Root Mean Square Residual (SRMR), which should be below 0.08; and the Root Mean Square Error of Approximation (RMSEA), ideally less than 0.08 and preferably below 0.06.

### 4.3 Research sample

The data collection was conducted in the form of a questionnaire survey over a period of three weeks from 7 January 2024 to 25 January 2024 and informed written consent was obtained from all participants and the research was approved by the Research Ethics Committee of Masaryk University. The survey was addressed to customers of individual providers of mobile electronic communications services in Slovakia, i.e. customers of Orange Slovensko, a.s., O2 Slovakia s.r.o., Slovak Telekom, a.s. and SWAN Mobile, a.s. Respondents were obtained through the social network Facebook, which offers public groups of individual Slovak cities, which ensured a broad sample of respondents. All members of the respective groups were always contacted.

All of the above companies provide mobile services, voice messaging and mobile internet, as well as internet and TV services, thus providing a complete telecommunications service. The activities of the players in this market can be characterised as an oligopoly with cartel elements [77].

In 2022, there were 7 556 000 SIM cards active in Slovakia. Of these, over 32% belonged to Orange Slovensko, a.s., 31% to O2 Slovakia s.r.o., over 28% to Slovak Telekom, a.s. and 8% to SWAN Mobile, a.s. [119]. A total of 1001 respondents were obtained, 994 of whom indicated that they use a SIM card of one of the mobile operators in Slovakia. Therefore, seven responses from respondents who indicated that they were not SIM card users of any of the mobile operators operating in Slovakia were excluded from the sample. In addition, duplicates for each provider were removed. Two duplicates were found in the total sample of customers of Orange Slovensko, a.s., three in the case of O2 Slovakia s.r.o., seven in the case of Slovak Telekom, a.s. and two in the case of SWAN Mobile, a.s. All fourteen duplicates were removed, so the research sample consisted of 980 respondents.

The individual mobile operators were represented as follows: Orange Slovensko, a.s. almost 36%, O2 Slovakia s.r.o over 24%, Slovak Telekom, a.s. 32% and SWAN Mobile, a.s. over 7%. O2 Slovakia s.r.o. was the only company that did not achieve a satisfactory number of respondents. Therefore, the number of 241 respondents obtained from this company was set at the required 31%, while for the other companies the following samples were obtained by random selection: Orange Slovensko, a.s. with 252–32% respondents, Slovak Telekom, a.s. with 222–28% respondents and SWAN Mobile, a.s. with 67–8% respondents. This brings the total industry sample down to 782 respondents, which is the final number of the research sample. Random sampling was carried out in Microsoft Excel by generating random numbers from a set of admissible data obtained for each company. All demographic characteristics are presented in Table 3.

**Table 3:** Demographic characteristics of the research population and users of mobile services in Slovakia.

	Number in sample	% of sample	of total population
Mobile operator			
Orange Slovensko, a.s.	252	32%	32%
O_2_ Slovakia s.r.o.	241	31%	31%
Slovak Telekom, a.s.	222	28%	28%
SWAN Mobile, a.s. (4ka)	67	8%	8%
Gender			
Woman	526	67%	51.1%
Man	256	33%	48.9%
Age			
Productive (18–64)	735	94%	67.03%
Post-productive (65 and over)	47	6%	17.05%
Pre-productive (0–18)	0	0%	15.92%
Status			
Employed	488	62%	40.2%
Student	107	14%	11.17%
Pensioner	63	8%	19.39%
Self-employed	53	7%	21.29%
On maternity/parental leave	50	6%	1.33%
Unemployed	13	2%	4.25%
Other	8	1%	2.37%
Education			
Higher Education	396	51%	18.38%
Secondary school with diploma	307	39%	24.66%
Secondary school without diploma	30	4%	19.22%
Higher vocational	29	4%	4.91%
Basic	10	1%	16.97%
None/ Unfinished	10	1%	11.72%
Marital status			
Single	357	46%	44.39%
Married	315	40%	40.05%
Divorced	92	12%	8.16%
Widowed	18	2%	7.04%
Residence			
100 000 and more	386	49%	2.09%
to 4 999	110	14%	46.96%
50 000–99 999	102	13%	10.53%
20 000–49 999	100	13%	22.28%
5 000–19 999	84	11%	18.13%

Source: own research according to touchIT, s.r.o. [120]; Scitanie.sk [105]

The characteristics of the respondents indicate that the sample is representative only in terms of the mobile operator they use and their marital status. However, the sample is skewed towards women, individuals of working age, employees with university degrees, and residents of large cities, making it non-representative in other demographic aspects.

## 5 results

First, the data were tested for collinearity using the correlation matrix and VIF. The highest values in the correlation matrix appeared between the variables PV1 and PV2 and CS1 and CS2. The correlation between them was just above the lowest threshold and the correlation coefficients were 0.854 and 0.847. The lowest VIF value achieved between the variables was 1.337 and the highest for the mentioned variables PV2 (5.32) and CS1 (5.56). Due to the fact that the VIF values of both these variables were significantly lower than 10, all the variables studied can be considered as non-collinear.

Cronbach alpha was used to assess the internal consistency of the data. The latent variables in the model reach the desired value, although the image variable is just above the minimum threshold of acceptability with a value of 0.676, and conversely, the satisfaction variable is just below the maximum threshold of acceptability with a value of 0.926. Nevertheless, the Cronbach alpha values of all latent variables can be considered acceptable (see Table 4 for more details), also considering the pairwise correlations of the variables.

**Table 4:** Cronbach alpha.

Variable	Cronbach alpha
Image	0.676
Customer expectations	0.814
Perceived quality	0.821
Perceived value	0.876
Customer satisfaction	0.926
Customer loyalty	0.851

Source: own research

All the variables meet the above conditions for confirming the discriminant validity of AVE, although the image variable only just does (see Table 5 for details). Convergent validity was clearly achieved as all standardized regression coefficients show a value greater than 0.5 (see Table 6 for details).

**Table 5:** Tests of discriminant validity and reliability of constructs in the model.

Variable	AVE	CR
Image	0.405	0.899
Customer expectations	0.593	0.939
Perceived quality	0.488	0.920
Perceived value	0.716	0.973
Customer satisfaction	0.806	0.980
Customer loyalty	0.663	0.961

Source: own research

**Table 6:** Regression coefficients of the measurement model.

Latent variable	Factor	Standardized regress coefficient	Non-standardized regress coefficient
Image	IM1	0.654	1.000
	IM2	0.678	1.031
	IM3	0.578	0.880
Customer expectations	EXP1	0.750	1.000
	EXP2	0.788	1.131
	EXP3	0.777	1.030
Perceived quality	PQ1	0.770	1.000
	PQ2	0.756	1.077
	PQ3	0.717	1.040
	PQ4	0.700	1.003
	PQ5	0.529	0.809
Perceived value	PV1	0.891	1.000
	PV2	0.957	1.063
	PV3	0.666	0.717
Customer satisfaction	CS1	0.934	1.000
	CS2	0.903	1.047
	CS3	0.856	0.932
Customer loyalty	CL1	0.831	1.000
	CL2	0.917	1.156
	CL3	0.681	0.916

Source: own research

All the latent variables also meet the reliability (CR) requirements as they exceed the value of 0.7 by a large margin (see Table 5 for details). Based on these tests, it can be concluded that the measurement model is both valid and reliable.

Table 7 clearly shows the individual latent variables created as estimated from the individual observed variables.

**Table 7:** Latent variables.

	Estimate	Std. Err.	z-value	P(>|z|)	Std. lv	Std. all
Image						
IM1	1.000				0.923	0.654
IM2	1.031	0.069	14.897	0.000	0.951	0.678
IM3	0.880	0.067	13.203	0.000	0.812	0.578
Customer expectations						
EXP1	1.000				0.742	0.750
EXP2	1.131	0.060	18.986	0.000	0.840	0.788
EXP3	1.030	0.055	18.879	0.000	0.764	0.777
Perceived value						
PV1	1.000				1.486	0.891
PV2	1.063	0.026	40.686	0.000	1.579	0.957
PV3	0.717	0.033	21.948	0.000	1.066	0.666
Perceived quality						
PQ1	1.000				1.041	0.770
PQ2	1.077	0.050	21.334	0.000	1.120	0.756
PQ3	1.040	0.052	20.126	0.000	1.082	0.717
PQ4	1.003	0.051	19.593	0.000	1.044	0.700
PQ5	0.809	0.056	14.414	0.000	0.842	0.529
Customer satisfaction						
CS1	1.000				1.314	0.934
CS2	1.047	0.024	43.669	0.000	1.376	0.903
CS3	0.932	0.025	37.560	0.000	1.224	0.856
Customer loyalty						
CL1	1.000				1.346	0.831
CL2	1.156	0.036	32.206	0.000	1.557	0.917
CL3	0.916	0.043	21.076	0.000	1.234	0.681

Source: own research

In the structural part, a model was constructed, the tests of which were more than satisfactory and the model below can be considered as validated. The respective tests reached the following values: CFI = 0.955, TLI = 0.946, SRMR = 0.038 and RMSEA = 0.061. The resulting values of the structural model are presented in Table 8.

**Table 8:** Structural model output values.

	Estimate	Std. Err.	z-value	P(>|z|)	Std. lv	Std. all
Customer expectations						
Image	0.336	0.042	8.045	0.000	0.418	0.418
Perceived quality						
Customer expectations	0.200	0.058	3.454	0.001	0.143	0.143
Image	0.903	0.070	12.870	0.000	0.800	0.800
Perceived value						
Perceived quality	0.991	0.066	15.092	0.000	0.694	0.694
Customer expectations	-0.183	0.081	-2.255	0.024	-0.091	-0.091
Customer satisfaction						
Perceived quality	0.397	0.096	4.154	0.000	0.314	0.314
Perceived value	0.494	0.027	17.974	0.000	0.559	0.559
Image	0.183	0.100	1.832	0.067	0.128	0.128
Customer expectations	0.037	0.045	0.822	0.411	0.021	0.021
Customer loyalty						
Customer satisfaction	0.931	0.044	21.366	0.000	0.909	0.909
Image	0.036	0.057	0.627	0.530	0.024	0.024

Source: own research

The results show that hypotheses H1 and H5 were not confirmed and hypothesis H2 was only confirmed at a lower level of significance (p = 0.1). The remaining hypotheses were confirmed at the standard level of significance (p = 0.5). Thus, it was possible to confirm a statistically significant positive relationship between image and customer satisfaction, image and customer expectations and image and perceived quality. It was also possible to show a statistically significant negative relationship between customer expectation and perceived value and a positive relationship between customer expectation and perceived quality and customer expectation and customer satisfaction (at a lower level of significance). It was also possible to show a statistically significant positive relationship between perceived quality and perceived value and between perceived quality and customer satisfaction. Finally, it was possible to show a statistically significant positive relationship between perceived value and customer satisfaction and customer satisfaction and customer loyalty.

## 6 discussion

The relationships between image and customer satisfaction, image and customer expectations, customer expectations and perceived quality, perceived quality and perceived value, perceived quality and customer satisfaction, perceived value and customer satisfaction, and customer satisfaction and customer loyalty are well-established in the literature, including within the telecommunications industry and complex models (see [7, 9, 15, 86]). These relationships have remained consistent even after the Covid-19 pandemic in the mobile telecommunications sector in Slovakia.

However, another positive relationship confirmed in the model is between image and perceived quality. This relationship has been consistently demonstrated in complex models, notably in the research of Strenitzerová & Gaňa [15] within the Slovakian market. While previously shown for a single firm, suggesting relevance to a specific segment, it aligns with findings in broader market studies (Slovakia). This indicates the relationship is typical across major telecommunication companies, rather than confined to a smaller subset. Moreover, the directionality from image to perceived quality has been consistently supported, consistent with findings in Strenitzer & Gaňa [15] and Gluhović [88], as opposed to vice versa (cf. [84, 86, 87]). Thus, this relationship holds true in the mobile telecommunications sector, particularly in markets similar to Slovakia in terms of size and development.

Another notable relationship demonstrated is between customer expectations and perceived value, historically found to be positive in prior research, including studies conducted in the Slovak market (see [7, 9, 15, 85]). However, in our research, this relationship shows a negative correlation. This discrepancy with previous findings suggests a shift in customer perceptions following the Covid-19 pandemic in the relevant market.

A negative relationship between these variables indicates that as expectations rise, perceived value decreases, and vice versa—a decrease in expectations leads to an increase in perceived value. This divergence may stem from customers’ increasingly critical evaluation of the industry, perceiving services as highly similar or lacking differentiation among mobile operators’ offerings in the market [81]. Consequently, customers perceive the value of mobile services as relatively low, while expectations for service delivery remain high. This observation aligns with the assertion by Chen [121] that customer expectations may not align with perceived value, reflecting a disconnect in customer perception and market reality.

Interestingly, two relationships in our research were found to be statistically insignificant, indicating unconfirmed associations. Specifically, these include the relationships between image and customer loyalty, and between customer expectations and customer satisfaction. These findings contrast with previous studies by Türkyilmaz & Ozkan [7], Türkyilmaz et al. [9], Ali et al. [53], Mohd Jamil et al. [85], Strenitzer & Gaňa [15], and Dam & Dam [46], where these relationships were confirmed.

The lack of confirmation regarding the relationship between image and customer loyalty aligns with the findings of Yilmaz & Ari [84] and Diputra & Yasa [86]. Similarly, the absence of a relationship between customer expectations and customer satisfaction is consistent with research by Johnson et al. [89], Martensen et al. [48], and Johnson et al. [90]. These results suggest a notable shift in the relationships between these factors possibly attributable to the Covid-19 pandemic, reflecting changing customer behaviors and expectations in the mobile telecommunications sector.

Interestingly, our research results diverge from those of Strenitzer & Gaňa [15], conducted in the same market. This discrepancy suggests that the situation observed in one firm (and its relevant segment of the market) may differ from the broader market context. Alternatively, it is plausible that there has been an overall change in market dynamics over the approximately six-year gap between the surveys, potentially influenced by the Covid-19 pandemic.

The hypothesis that the pandemic or its aftermath may be influencing these changes is supported by research conducted by Pollák et al. [122], Markovič et al. [123], and indirectly, in other sectors and countries, by Song et al. [22], Al-Hattami et al. [23], Sakas et al. [20], and Susanto et al. [21]. These studies provide evidence of significant shifts in consumer behavior and market conditions following the pandemic, which could reasonably extend to the mobile telecommunications sector as well.

## 7 conclusion

Our research successfully developed a model of customer satisfaction in the mobile telecommunications industry, elucidating essential variables for satisfaction modeling. Interestingly, most relationships in the model remained stable after the Covid-19 pandemic. However, notable changes were observed compared to the model by Strenitzer & Gaňa [15] for the same industry and country.

Firstly, we confirmed a positive relationship between image and perceived product quality across the entire Slovak mobile telecommunications market. Importantly, this relationship confirms the direction of influence from image to perceived product quality, rather than vice versa. Secondly, we identified a significant change in the relationship between customer expectations and perceived customer value. Contrary to previous research in mobile telecommunications, our findings revealed a negative relationship between these variables. This represents a noteworthy divergence from established models of customer satisfaction in the industry.

In conclusion, our research identified two relationships that were not confirmed: between image and customer loyalty, and between customer expectations and customer satisfaction. This suggests a significant impact of the Covid-19 pandemic on the mobile telecommunications industry in our country.

Given the sector’s characteristics, where customer retention and loyalty are critical for business success, it is paramount for managers to prioritize customer satisfaction. This factor exerts a dominant and direct influence on customer loyalty. Furthermore, there is a clear need for long-term strategies to reshape customer expectations regarding services offered. This includes differentiation from competitors and enhancing service quality to not only raise customer expectations but also ensure they perceive high value in the product due to its superior quality. These efforts are crucial for sustaining customer satisfaction amidst evolving market conditions.

In the short term, managers may consider lowering prices to enhance the perceived value of their services, potentially gaining a competitive edge. However, this strategy could also lead to reduced revenue if it fails to attract enough new customers, especially given market conditions and existing switching barriers. Therefore, focusing on long-term strategies to enhance competitiveness, such as improving product quality and differentiation, is essential. Nevertheless, increasing product quality carries the risk of higher costs, which could impact firm performance.

As these findings pertain specifically to the Slovak market, further research in other economies, both transitional and developed, is recommended to validate the relationships identified in our satisfaction model. Additionally, future research should delve into the underlying causes behind these results. This includes not only confirming the influence of the Covid-19 pandemic but also uncovering other factors that contribute to the observed relationships within market dynamics or company operations.

Limitations of this study include the focus on modelling customer satisfaction within the mobile telecommunications submarket, which itself is a subset of the broader telecommunications sector and the national economy (specifically, Slovakia). While there is a hypothesis about the model’s applicability to similar transitional economies, this hypothesis could not be tested in this research, representing a significant limitation.

Another limitation pertains to the sample used in this study, which, while representative in terms of mobile operator choice and marital status, may not fully capture other demographic and socioeconomic factors. Future research would benefit from a more comprehensive and larger sample size to enhance the generalizability of the findings. Additionally, the complexity of the questionnaire and the number of variables assessed in this study suggest that a larger sample size could provide more robust insights into the relationships examined.

## Supporting information

S1 File Inclusivity in global research. (DOCX)

S2 File Questionnaire. (DOCX)
