# Supplementary material for: The customer satisfaction model in the mobile telecommunications sector after Covid-19 pandemic
Source: PLoS One. 2025 Jan 27;20(1):e0317093. doi: 10.1371/journal.pone.0317093 (PMC11771900; doi:10.1371/journal.pone.0317093)
Supplement: S2 File — (DOCX) [file pone.0317093.s002.docx]

**Questionnaire**

As a research participant, I hereby agree to the anonymous processing of my answers to the questions in the questionnaire below and I also agree to the publication of the relevant results (in anonymized and aggregated form together with the other respondents) by the authors of the questionnaire. (Y/N)

| Variable | Question from the questionnaire | Answer options |
| --- | --- | --- |
|  | Do you use a sim card from a mobile operator in Slovakia? | 1 Yes  2 No |
| Questions to determine satisfaction with a mobile operator | | |
| IM1 | Mobile operator innovates services | 1 - strongly disagree  2 - disagree  3- rather disagree  4- neither agree nor disagree  5- rather agree  6 - agree  7- strongly agree |
| IM2 | Employees have a professional approach to me |  |
| IM3 | Mobile operator engages in social responsibility activities |  |
| EXP1 | I expect quality of service provided by the mobile operator |  |
| EXP2 | I expect my needs to be met based on the expectations I have of the mobile operator |  |
| EXP3 | I expect the mobile operator's staff to be prompt and ready to solve problems with the service provided |  |
| PQ1 | The mobile operator provides quality voice and data services |  |
| PQ2 | The mobile operator provides a quality mobile network and coverage |  |
| PQ3 | The mobile operator has a quality mobile application |  |
| PQ4 | The mobile operator provides quality customer support |  |
| PQ5 | The mobile operator has a dense network of branches |  |
| PV1 | The mobile operator's services are affordable for me |  |
| PV2 | The mobile operator offers good value for money |  |
| PV3 | The price of mobile services with my operator is better than competitors |  |
| CS1 | Overall, I am satisfied with my mobile operator |  |
| CS2 | The mobile operator meets all my expectations |  |
| CS3 | Compared to possible ideal mobile services, I am satisfied with the ones I use |  |
| CL1 | I plan to purchase my mobile operator's services again in the future |  |
| CL2 | I would recommend my mobile operator to family, friends and acquaintances |  |
| CL3 | I will continue to use my mobile operator's services even if they increase prices |  |
| Demographic characteristics | | |
| I. | Which mobile operator do you use? | 1 Orange Slovensko, a.s.  2 O2 Slovakia s.r.o.  3 Slovak Telekom, a.s.  4 SWAN Mobile, a.s. (4ka) |
| II. | What is your gender? | 1 Man  2 Woman |
| III. | What is your age? | 1 Pre-productive (0 - 14)  2 Productive (15 - 64)  3 Post-productive (65 and over) |
| IV. | Are you currently: | 1 Employee  2 Unemployed  3 Student  4 Pensioner  5 On maternity/parental leave  6 Self-employed  7 Other |
| V. | What is your highest level of education? | 1 None/ not yet completed  2 Basic  3 Secondary without diploma  4 Secondary school with diploma  5 Higher vocational  6 Higher education |
| VI. | What is your marital status? | 1 Single  2 Married  3 Divorced  4 Widowed |
| VII. | Do you live in a village/town that has: | 1 to 4 999  2 5 000 - 9 999  3 10 000 - 19 999  4 20 000 - 49 999  5 50 000 - 99 999  6 100 000 and more |
